# Utilization of prevention of mother-to-child transmission (PMTCT) services among pregnant women in HIV care in Uganda: a 24-month cohort of women from pre-conception to post-delivery

**DOI:** 10.1186/s13104-018-3304-y

**Published:** 2018-03-22

**Authors:** Rhoda K. Wanyenze, Kathy Goggin, Sarah Finocchario-Kessler, Jolly Beyeza-Kashesya, Deborah Mindry, Josephine Birungi, Mahlet Woldetsadik, Glenn J. Wagner

**Affiliations:** 1 Department of Disease Control and Environmental Health, Makerere University School of Public Health, Kampala, Uganda; 2 Health Services and Outcomes Research, Children’s Mercy Hospital and University of Missouri, Kansas City, USA; 3 Schools of Medicine and Pharmacy, University of Missouri, Kansas City, USA; 4 Department of Family Medicine, University of Kansas Medical Center, Kansas City, USA; 5 Mulago Hospital Department of Obstetrics and Gynaecology, Makerere University College of Health Sciences, Kampala, Uganda; 6 Los Angeles Center for Culture and Health, University of California, Los Angeles, USA; 7 The AIDS Support Organization, Kampala, Uganda; 8 RAND Corporation, Santa Monica, USA

**Keywords:** PMTCT, Service utilization, HIV infected women in care

## Abstract

**Objective:**

We assessed the uptake of prevention of mother-to-child transmission (PMTCT) services in a cohort of HIV infected women in care at The AIDS Support Organization Jinja and Kampala in Uganda, who were trying to conceive, over a period of 24 months, to inform the strengthening of PMTCT service access for women in care.

**Results:**

Of the 299 women 127 (42.5%) reported at least one pregnancy within 24 months; 61 women (48.0%) delivered a live child. Of the 55 who had a live birth at the first pregnancy, 54 (98.2%) used antenatal care (ANC) starting at 15.5 weeks of gestation on average and 47/49 (95.9%) delivered at a health facility. Excluding miscarriages, 54 (98.2%) received ARVs during pregnancy. Of the 49 live births with post-delivery data, 37 (75.5%) tested the infant for HIV. 79 of the 127 (68.7%) spoke with providers about childbearing. Communication with providers was associated with ANC use (65.8% vs. 41.7%; p = .015). Despite the high rate of miscarriages and late ANC start, this study shows very high uptake of PMTCT services among PLHIV in care and their infants. Improved provider–client communication could enhance ANC attendance and PMTCT outcomes among HIV infected women in care.

## Introduction

Access to prevention of mother-to-child transmission of HIV (PMTCT) services is critical to elimination of vertical transmission of HIV [1]. Several countries, including Uganda have made remarkable progress towards elimination of vertical transmission of HIV [2, 3]. However, some bottlenecks remain in ensuring wider access to and utilization of services across the PMTCT cascade [1–3]. HIV infected women who are unaware of their HIV status or are aware but not yet in care at conception pose a major challenge to the provision of effective PMTCT services [2, 3]. Pregnant women of unknown HIV status must receive an HIV diagnosis and if positive, a referral to PMTCT services amidst multiple competing priorities. Those who are diagnosed with HIV but not in care must quickly overcome their initial barriers to linkage to care and ensure they access PMTCT services [4]. Further, delay to initiate and sustain antenatal care (ANC) and low facility based deliveries create additional challenges for PMTCT service uptake [5–7].

Many countries have made progress towards the UNAIDS 90-90-90 targets, diagnosis of at least 90% of the HIV infected individuals, initiation of antiretroviral therapy (ART) for 90% of those diagnosed and achieving viral suppression for 90% of those initiating ART [3, 8]. This provides an opportunity to integrate antenatal care (ANC) services including early and sustained PMTCT services for PLHIV in care [9, 10]. Since women in care frequently interface with the health system, they would ideally initiate ANC earlier, attend the required number of visits, and deliver at health facilities all of which are associated with better outcomes. Women who plan their pregnancies and intentionally conceive while in care should be even more vigilant with accessing PMTCT services.

Unfortunately, the integration of SRH services in HIV care is a long-standing challenge despite the high fertility among PLHIV in sub-Saharan Africa [11–17]. In this analysis, we assessed the uptake of PMTCT services in a cohort of HIV infected women in care who were trying to conceive to inform the strengthening of PMTCT service access for women in care.

## Main text

### Methods

#### Study design

This study was conducted as part of a larger cohort study of safer conception among PLHIV in care with fertility intentions.

#### Study setting

The study was conducted at two sites within The AIDS Support Organization (TASO) in Uganda [18, 19]. TASO Kampala located next to the Mulago National Referral Hospital and TASO Jinja within the Jinja Regional Referral Hospital in Jinja district. At the time, TASO Mulago had > 8300 active clients in care while TASO Jinja had > 7600 clients. TASO integrated family planning services and provided ART to HIV infected pregnant women on site. Women were referred to Mulago and Jinja hospitals for ANC and delivery services, although TASO provided pregnancy monitoring and related services onsite.

#### Participant selection, eligibility screening, and interviews

The primary study from which these data were derived included 400 PLHIV in care (including 299 HIV-infected women) and was powered to evaluate the use of use of safer conception methods [18]. The current analysis includes 127 women who reported at least one pregnancy within the 24 months of follow-up. Clients were eligible for study participation if they were ≥ 18 years, married or in a committed heterosexual relationship, and intended to conceive a child within 24 months. The cohort was recruited between May and October of 2013. A brief screening was conducted by the triage personnel. Those who were likely eligible were referred to the research coordinator for detailed screening and consent procedures before the interview. Follow-up interviews were conducted at 6-month intervals for 24 months. If the participant delivered a child, a survey was scheduled 1 month after the delivery.

#### Measures

Interviews were administered in Luganda, the most common language in the study setting, using computer-assisted personal interview software. The measures were developed by the study team and have been described elsewhere [18]. Measures included basic socio-demographic characteristics, fertility desires and intentions, and childbearing history including the number of living children, history ANC attendance, and access to ARVs by the woman and exposed infant. Women were asked if they attended ANC and what gestation week they had their first visit, if they received ARVs prior to, during pregnancy or after pregnancy. The CD4 data was obtained through a review of patient records. Other variables included venue of the delivery (including health facility, with traditional birth attendants or at home), receipt of post-natal care (PNC), and infant services including ARVs for prophylaxis, early infant diagnosis (EID), EID timing, and the outcome of the test (positive, negative, did not get results).

#### Data analysis

The main outcomes were ANC attendance and receipt of PMTCT ART. Descriptive statistics were conducted to describe characteristics of study participants, and access to ANC and PMTCT services by the women and exposed infant. With near universal access to ANC and PMTCT ART among women who had live births, we did not examine the correlates of ANC and PMTCT ART. This paper is thus largely a descriptive analysis of PMTCT service uptake. We however we conducted bivariate statistics of the correlates of child HIV testing and provider–client communication (Chi Square tests, 2-tailed independent t-tests) were used to examine the baseline correlates. A p value of < .05 was used to represent a significant difference. SPSS version 15.0 was used for data analysis.

### Results

Overall, 299 HIV-infected women with fertility intentions were enrolled, 127 (42.5%) of whom had at least one pregnancy during the study (Table 1). Of the 127, 18 (14.2%) had two or more pregnancies within 24 months. Repeat pregnancies were reported by one of the 55 women who had a live birth in their first pregnancy, compared to 16 of 69 (23.2%) who had stillbirths and/or abortions/miscarriages at the first pregnancy (the remaining 3 participants were still pregnant at the end of the study and we were unable to ascertain their outcome). Of the 17 people who had more than one pregnancy, 7 had a successful pregnancy on their second or third try during the course of the study. Overall, 61 participants delivered a child during the study. The data reported on pregnancy care processes and outcomes in the remainder of the paper is based only on data from the first pregnancy reported by the participant. Whereas the occurrence of the second pregnancies and their outcomes was recorded, the comprehensive ANC and service utilization was not documented for subsequent pregnancies.

**Table 1:** Socio-demographic characteristics, pregnancy care processes, and outcomes among female participants who had a pregnancy during the course of the study

Variable	N/mean N = 127^a^	Percentage/SD
Age (years)	31.7	SD = 6.0
Education (any secondary schooling)	51	40.2%
Married to partner	46	36.2%
HIV status of partner		
Negative	39	30.7%
Positive	45	35.4%
Unknown	43	33.9%
Partner is aware of respondent’s HIV status	94	74.0%
Baseline CD4 count	449	SD = 303
Months since HIV diagnosis	63.4	SD = 50.9
On ART at baseline	78	61.4%
Number of children prior to enrollment	2.5	SD = 1.5
Communicated with provider about desire to have a child	79/115	68.7%
Received ANC during pregnancy	73/121	60.3%
Received PMTCT ART during pregnancy	103/124	83.1%
Outcome of first pregnancy during study		
Successful delivery	55	43.3%
Miscarriage	67	52.8%
Induced abortion	1	.8%
Still birth	1	.8%
Still pregnant at end of study (outcome undetermined)	3	2.4%

^a^The percent values represent the percentages of the participants out of the total sample

#### ANC care and delivery

Among 73 women who used ANC at least once, care started at an average of 15.5 weeks of gestation (range 4–36 weeks). Among the 55 who delivered a live child, 54 (98.2%) used ANC, compared to 18 of 64 (28.1%) who did not successfully give birth (5 others who did not deliver a child had missing data) (p = .000). Miscarriages were experienced at an average of 13.0 weeks of gestation (71.4% within first trimester), which may explain why so many did not get to the point of engaging in ANC. Overall, 47/49 (95.9%) delivered at a health facility (6 had missing data).

#### Received ART (PMTCT services)

Overall, 103 (81.1%) received ART during pregnancy, including 72 (69.9%) who were on ART prior to conception and 31 (30.1%) who initiated ART during pregnancy—started at a mean of 11.2 (SD = 6.6; median = 12) weeks of gestation (range 1–24 weeks). 54 of the 55 (98.2%) women who gave birth used ART during pregnancy, compared to 47 of 67 whose pregnancy did not end in a successful delivery (2 had missing data) (p = .000). Of those on ART, 17% (17/100) reported missed doses (3 missing data) at any time during the pregnancy.

#### Receipt of PMTCT infant services

Of these 49 women with post-delivery data, 37 (75.5%) reported that the infant had been tested for HIV, of whom 28 were reportedly HIV-negative, while 9 (24.3%) did not return for the results. None reported that their child tested positive. All but one of the children were tested within first 2 months of life, and the other was tested at week 12. There were no significant correlates of child HIV testing among demographics, HIV characteristics, and psychosocial variables. 47 (95.9%) of the 49 respondents reported that the infant was given nevirapine prophylaxis at birth. Further, 36/48 (75.0%) of the infants received cotrimoxazole (1 missing). All 49 participants breastfed and 2 of these reported mixed feeding.

#### Communication with provider about childbearing

79/127 (68.7%; 12 missing) spoke with providers about childbearing prior to pregnancy. Communication with providers was associated with ANC use (65.8% vs. 41.7%; Chi square = 5.9; p = .015), and use of PMTCT (87.3% vs. 72.2%; p = .047), but not the likelihood of a successful delivery (46.8% vs. 36.1%; p = .288) or whether or not the child was tested for HIV among those who had a live birth (78.9% vs. 61.5%; p = .214).

### Discussion

These data show high uptake of ANC and PMTCT services with near universal ANC attendance (98%), delivery at health facilities (96%), and receipt of ARVs (98%) during pregnancy. The ANC attendance and uptake of ARVs during pregnancy in this population is close to the national level indicators (95 and 93%, respectively) [3]. However, the ANC attendance started late and could be improved. Close to two-thirds of the women on ART started treatment prior to pregnancy, which is also not surprising—the percentage of HIV+ women on ART prior to pregnancy has grown over the years due to expanded access to Option B+, and many women on ART having repeat pregnancies [3]. This percentage will likely continue to grow with scale-up of Option B+ and is indeed a desirable scenario as it reduces the challenges of initiation of ART among newly diagnosed individuals [2, 3].

Despite the poor pregnancy outcomes, these data indicate that ANC and PMTCT service uptake could be enhanced by having more PLHIV in care prior to conception even for persistently challenging indicators such as facility based deliveries (estimated at 57% in the general population in Uganda vs. 96% in this study) [20, 21]. However, more attention is needed to enhance provider–client communication and earlier ANC attendance. EID within 8 weeks of childbirth has also been a difficult indicator, with current estimates at < 50% [3]. However, almost three quarters of the participants in this sample had their infants tested for HIV, with almost all of the infants tested within 8 weeks. The high testing is however dampened by about a quarter of those testing not receiving results. Some studies have reported challenges with infant testing even for PLHIV in care, including fear of HIV diagnosis among their infants, among other challenges [22]. Receipt of ARVs for prophylaxis among exposed infants, was near universal probably due to very high facility based deliveries. Failure to deliver at health facilities has been associated with low ARV prophylaxis access among infants [22].

About two-thirds of PLHIV discussed childbearing with their providers prior to conception, which may have positively impacted on their ANC attendance. There is however room for further improvements in provider communication coupled with comprehensive information to address some of the highlighted gaps including receipt of EID results and cotrimoxazole uptake.

### Conclusion

Although not primarily designed to assess uptake of PMTCT services, this study shows very high uptake of PMTCT services, ANC attendance, and facility based deliveries among HIV infected women in care, a potential indicator of improved MCH and PMTCT outcomes with increased enrollment into HIV care of PLHIV. These benefits could be enhanced with improved client-provider communication and earlier ANC attendance. More effort is also required to further improve EID and cotrimoxazole uptake among infants.

### Limitations

This study was not primarily designed to assess uptake of PMTCT services among PLHIV in care and did not have sufficient power to assess the correlates of service uptake. Similarly, service uptake data was not available on all pregnancies particularly those that conceived later into the study. However, the study provides pointers to the potentially beneficial MCH and PMTCT effects arising from increased enrollment of PLHIV in care, which can be further assessed through larger studies.

## Abbreviations

ANC: antenatal care
ART: antiretroviral therapy
eMTCT: elimination of mother-to-child transmission of HIV
EID: early infant diagnosis
PLHIV: people living with HIV
PMTCT: prevention of mother-to-child transmission
TASO: The AIDS Support Organization
